# Educational outreach visits to improve venous thromboembolism prevention in hospitalised medical patients: a prospective before-and-after intervention study

**DOI:** 10.1186/1472-6963-13-398

**Published:** 2013-10-08

**Authors:** Jed Duff, Abdullah Omari, Sandy Middleton, Elizabeth McInnes, Kim Walker

**Affiliations:** 1 St Vincent’s Private Hospital Sydney, Victoria Street, 2010, Darlinghurst, NSW, Australia; 2 National Centre for Clinical Outcomes Research, Australian Catholic University, Sydney, Australia; 3 University of Tasmania School of Nursing & Midwifery, Education Centre, 1 Leichhardt St, 2010, Darlinghurst, NSW, Australia; 4 Nursing Research Institute, St Vincent’s & Mater Health Sydney- Australian Catholic University, St Vincent’s Hospital, Victoria Street, 2010, Darlinghurst, NSW, Australia

**Keywords:** Educational outreach visit, Implementation science, Venous thromboembolism prevention

## Abstract

**Background:**

Despite the availability of evidence-based guidelines on venous thromboembolism (VTE) prevention clinical audit and research reveals that hospitalised medical patients frequently receive suboptimal prophylaxis. The aim of this study was to evaluate the acceptability, utility and clinical impact of an educational outreach visit (EOV) on the provision of VTE prophylaxis to hospitalised medical patients in a 270 bed acute care private hospital in metropolitan Australia.

**Methods:**

The study used an uncontrolled before-and-after design with accompanying process evaluation. The acceptability of the intervention to participants was measured with a post intervention survey; descriptive data on resource use was collected as a measure of utility; and clinical impact (prophylaxis rate) was assessed by pre and post intervention clinical audits. Doctors who admit >40 medical patients each year were targeted to receive the intervention which consisted of a one-to-one educational visit on VTE prevention from a trained peer facilitator. The EOV protocol was designed by a multidisciplinary group of healthcare professionals using social marketing theory.

**Results:**

Nineteen (73%) of 26 eligible participants received an EOV. The majority (n = 16, 85%) felt the EOV was effective or extremely effective at increasing their knowledge about VTE prophylaxis and 15 (78%) gave a verbal commitment to provide evidence-based prophylaxis. The average length of each visit was 15 minutes (IQ range 15 to 20) and the average time spent arranging and conducting each visit was 92 minutes (IQ range 78 to 129). There was a significant improvement in the proportion of medical patients receiving appropriate pharmacological VTE prophylaxis following the intervention (54% to 70%, 16% improvement, 95% CI 5 to 26, p = 0.004).

**Conclusions:**

EOV is effective at improving doctors’ provision of pharmacological VTE prophylaxis to hospitalised medical patients. It was also found to be an acceptable implementation strategy by the majority of participants; however, it was resource intensive requiring on average 92 minutes per visit.

## Background

Venous thromboembolism (VTE) is a common and potentially devastating complication of hospitalisation. Failure to provide appropriate VTE prophylaxis can result in serious adverse outcomes including symptomatic deep vein thrombosis (DVT) or pulmonary embolism (PE), post-thrombotic syndrome, chronic pulmonary hypertension, recurrent VTE, or fatal PE. Each year in the United States there are an estimated one million cases of VTE resulting in approximately 300,000 deaths annually [1]. Together, the combined morbidity and mortality associated with this disease process result in an estimated economic burden to the nation of $1.5 billion/year [2].

People who are hospitalised with acute medical illness are particularly at risk of VTE. Without effective prophylaxis 10-20% of medical patients will develop an objectively diagnosed VTE which, in turn, has the potential to result in fatal PE. Within the acute patient population, fatal PE accounts for 10% of all deaths making it the single most preventable cause of hospital related mortality [3]. Contrary to common held perceptions, a significant proportion of these deaths occur in the medical patient population. A retrospective evaluation of 6833 autopsies found that 80% of the fatal PEs occurred in medical (nonsurgical) patients [4].

VTE in hospitalised medical patients is preventable; evidence-based guidelines recommend the use of low molecular weight heparin, low dose unfractionated heparin, or fondaparinux for patients deemed to be at increased risk of VTE [5,6]. Risk factors for VTE in medical patients include active cancer, previous VTE, reduced mobility, known thrombophylic condition, increased age, heart and/or respiratory failure, myocardial infarction, ischaemia stroke, acute infection or rheumatologic condition, obesity, and ongoing hormonal treatment [5,6]. A number of tools have been developed and validated to aid in the assessment of VTE risk and help determine the onset, intensity, type, and duration of recommended prophylaxis [7-9].

Despite the widespread availability of evidence-based guidelines on VTE prevention hospitalised medical patients still receive suboptimal prophylaxis [10-13]. One international study, the ENDORSE study, found that only 40% of at risk medical patients (n = 37,356) were receiving the recommended prophylaxis [12,14]. Numerous strategies to improve VTE prevention in hospitalised patients have been studied with varying degrees of success [15-19]. The evidence suggests that active implementation strategies that engage the target population are more effective than passive strategies at changing clinician behaviour and improving prophylaxis rates [15,17,19].

An educational outreach visit (EOV) is an active implementation strategy that entails a structured one-to-one educational visit conducted in the clinical setting of the participant by a trained facilitator [20]. This intervention is also known as university-based educational detailing, academic detailing, and educational visiting [21]. An EOV is based on social marketing theory. It relies on the psychological principles of persuasion to influence clinician behaviour and promote evidence-based practices [22]. A Cochrane systematic review of this implementation strategy concluded that EOVs, alone or in combination with other interventions, are consistently effective at influencing prescribing practices [21]. There have been few studies, however, examining the clinical impact of EOVs on the provision of VTE prophylaxis to medical patients and no previous studies on its acceptability or utility.

## Methods

### Objective

To evaluate the acceptability, utility and clinical impact of an EOV on doctors’ provision of pharmacological VTE prophylaxis to hospitalised medical patients.

### Target population

The target population was doctors who regularly admit medical (nonsurgical) patients to the study site. Regular was defined as being in the top two quartiles of admitters which equated to a minimum of 40 admissions per year.

### Setting

The study site is a 270 bed acute care private hospital in Sydney, Australia. It provides services in all major fields of medicine and surgery with the exception of obstetric and paediatric care. The hospital has approximately 20,000 admissions annually, with approximately 30% admitted for acute medical illness. As is the case in most private hospitals in Australia, patients are cared for by consulting medical officers with minimal support from junior medical staff.

### Ethics

The study received ethics clearance from the St Vincent’s Human Research Ethics Committee (File number 11/051).

### Intervention

A vascular medicine physician with expertise in VTE prevention was recruited to the role of EOV facilitator and was responsible for arranging and conducting each visit. The facilitator followed a strict protocol which was collaboratively developed by a multidisciplinary team of healthcare professionals (see Table 1). A Cochrane systematic review [21] and social marketing literature [22-24] informed the development of the protocol.

**Table 1:** Educational outreach visit protocol

**EOV component**	**Element**
Planning the visit	Contact the target population by email, phone, or in person
	Negotiate a convenient time and location for the visit
	Reconfirm arrangements prior to the visit
	Discuss with the research team any recruitment difficulties
Setting the scene	Ensure appropriate space for the discussion
	Engage in small talk to place the participant at ease
	Explain the purpose of the visit
	Negotiate the session length (approximately 20 minutes)
	Introduce the four key messages and identify participants specific needs
Building trust, credibility and likability	Mention the key opinion leaders in support of the project
	List the project’s academic and clinical affiliations
	Highlight your own clinical expertise in the area
	Attempt to uncover personal similarities between participant and yourself
	Offer genuine praise where appropriate
Promoting two-sided communication	Ask open ended questions
	Use minimal encouragement techniques
	Paraphrase and reflect on the participants comments
	Anticipate and acknowledge controversial issues
	Overcome any objections and handle challenging responses
Delivering key message(s)	VTE is an important healthcare issue
	Assess individual patient risk
	Provide evidence-based VTE prophylaxis and patient education
	Monitor and reassess each patient during their hospital stay
Wrapping up	Reflect on the discussion
	Reiterate the key message(s) discussed
	Give the participant the printed resource material to keep
	Gain commitment to provide evidence-based prophylaxis
Providing follow-up	Follow-up via email, phone, or in person
	Fulfil any commitments made during the visit

The EOV facilitator and research team received training on social marketing and persuasive communication techniques in a two day workshop run by an independent not-for-profit organisation (the National Prescribing Service) with extensive experience in the use of EOVs for the promotion of the quality use of medicine in the Australian healthcare system.

The multidisciplinary group also developed the content to be delivered by the facilitator during the EOV. The content was limited to four key messages: 1) VTE is an important healthcare issue which results in significant mortality, morbidity and resource expenditure; 2) patients must have their risks assessed including clotting risk, bleeding risk, and contraindications to prophylaxis; 3) patients must receive appropriate prophylaxis based on their risk assessment; and 4) patients must be monitored for signs of VTE or prophylaxis related adverse events. A concise graphic educational resource was developed to accompany and reinforce the verbal message.

Two trial visits were conducted prior to the intervention period to identify potential issues and familiarise the facilitator with the protocol.

### Outcome measures and data collection

#### Acceptability

Acceptability was measured with post intervention participant and facilitator surveys. The participants’ survey and self-addressed envelope were given to the participants by the facilitator following the EOV. The survey contained eight questions in total; six questions related to the doctor’s beliefs about the effectiveness of the EOV at increasing knowledge and addressing concerns about VTE prophylaxis for medical patients. The remaining two questions asked participants how likely it was that they would participate in a program such as this in the future, and how likely it was that the intervention would influence their clinical practice. The EOV facilitator was also asked to complete a post intervention survey appraising the participants’ level of interest, participation and comprehension. All survey questions were answered on a five point likert scale.

#### Utility

Descriptive data on the practical application and utility of the intervention were recorded on a data collection form by the EOV facilitator. The information included the time and effort spent arranging the EOV, the time spent conducting the EOV, the number of interruptions and the time spent on them, the location of the EOV, the facilitator’s self-assessed adherence to the elements of the study protocol, and whether or not the participant committed to provide evidence-based prophylaxis.

#### Clinical impact

Clinical impact was assessed by auditing the proportion of medical patients receiving appropriate pharmacological VTE prophylaxis before and after the EOV intervention. The audits were conducted using an audit tool based on national VTE prevention guidelines [6]. A registered nurse trained in the use of the tool conducted each audit with expert adjudication from a consultant vascular physician when required. VTE prophylaxis was deemed appropriate if it conformed to the locally endorsed guideline with consideration given to individual risk status and contraindications. As per the guideline, patients were classified as high-risk based on the presence of one or more known risk factor (see Table 2). Contraindications to pharmacological prophylaxis included active bleeding; high risk of bleeding; severe hepatic disease; heparin induced thrombocytopenia; or current anticoagulation. The definition of appropriate includes prophylaxis prescribed when indicated; and prophylaxis not prescribed when contraindicated. The following exclusion criteria were used for patient selection: Planned or prior (previous 30 days) surgery on that admission; admitted for less than 24 hours; and inadequate documentation to complete a risk assessment.

**Table 2:** VTE risk factors & contraindications to prophylaxis

**Risk factors & contraindications**	**Pre intervention (n = 150)**	**Post intervention (n = 150)**	**P Value**
**High-risk of VTE**	126 (84)	116 (77)	0.14^
**Risk factors**			
Ischaemic stroke	7 (4.7)	3 (2)	0.19^
History of VTE	15 (10)	18 (12)	0.58^
Active cancer	4 (2.7)	4 (2.7)	1.0^
Decompensated heart failure	42 (28)	29 (19)	0.7^
Acute on chronic lung disease	10 (6.7)	10 (6.7)	1.0^
Age > 60 years and immobile	107 (71)	108 (72)	0.89^
Acute inflammatory disease	6 (4)	1 (0.7)	0.5^
Multiple additional risk factors	21 (14)	10 (6.7)	0.33^
**Additional risk factors**			
Immobility (<60 years)	1 (0.7)	1 (0.7)	0.98^
Familial history of VTE	1 (0.7)	0 (0)	0.31^
Oestrogen therapy	2 (1.4)	1 (0.7)	0.55^
Obesity	10 (6.8)	7 (4.7)	0.43^
Thrombophilia	1 (0.7)	0 (0)	0.313^
Active inflammation	6 (4.1)	2 (1.4)	0.09^
**Contraindications**	48 (32)	49 (32.7)	0.15^
Active bleeding	5 (3.3)	5 (3.3)	1.0^
High risk of bleeding	5 (3.3)	7 (4.7)	0.55^
Severe hepatic disease	1 (0.7)	0	0.31^
Heparin induced thrombocytopenia	0 (0)	0 (0)	
Current anticoagulation	41 (27.3)	38 (25.3)	0.69^
Other contraindication	0 (0)	2 (1.3)	0.15^

Percentages may not add up to 100 due to missing data. *SD* Standard Deviation; ^Chi-square.

### Sample size

The study was designed to detect a change in prescribing practice of 15% (from 50% to 65% appropriate prophylaxis). This estimate of effect was based on two previous studies which had used EOVs to improve VTE prophylaxis in the acute care setting [25,26]. A total sample size of 300 patients (150 pre and 150 post intervention) was necessary to power the study at 80% with a significance level of 5%.

### Data analysis

Data were entered into SPSS version 18 for analysis. Categorical data were summarised as number and percentage and contiguous data were summarised as median and interquartile (IQ) range. For comparisons between groups, the T test, or Mann–Whitney U test, was used for continuous variables (age, number of years post registration) and the Chi-square test was used for dichotomous variables (appropriate prophylaxis, risk factors, sex, specialty unit, admitting specialty). The difference in pharmacological prophylaxis rates before and after the intervention was calculated with 95% confidence intervals. The p value for statistical significance was set at <0.05.

## Results

### Characteristics of the target population

Of the 26 doctors who met the inclusion criteria 19 (73%) agreed to participate in the intervention and seven (27%) declined or were unavailable. The demographic characteristics of the target population are shown in Table 3. The median age of the participants was 54 years (IQ range 42–65) and their median number of years post registration was 30 years (IQ range 18–41). Fifteen (79%) were male and four (21%) female. The clinical specialties of the doctors were cardiology (n = 8, 42%); neurology (n = 4, 21%); nephrology (n = 1, 5.3%); medical oncology (n = 1, 5.3%); immunology/rheumatology (n = 2, 10%); thoracic medicine (n = 2, 10%); and gastroenterology (n = 1, 5.3%). There was no statistical difference in sex, number of years post registration, or specialty between doctors who received the intervention and those who declined or were unavailable.

**Table 3:** Characteristics of the target population

**Characteristics**		**Received the intervention (n = 19)**	**Declined or unavailable (n = 7)**	**P value**
**Age**	Median (IQ range)	54 (42–65)	N/A	
**Years post registration**		30 (18–41)	26 (23–33)	0.93*
**Sex**	Number (%)			0.18^
Male		15 (79)	7 (100)	
Female		4 (21)	0 (0)	
**Specialty**				0.32^
Cardiology		8 (42)	3 (43)	
Neurology		4 (21)	1 (14)	
Nephrology		1 (5.3)	0 (0)	
Medical oncology		1 (5.3)	0 (0)	
Immunology/ rheumatology		2 (10)	0 (0)	
Thoracic medicine		2 (10)	0 (0)	
Gastroenterology		1 (5.3)	3 (43)	

Percentages may not add up to 100 due to missing data. *IQ* Interquartile range; *N/A* not available; *Mann–Whitney U test; ^Chi-square.

### Characteristics of the audited patients

A total of 300 consecutive patients who met the inclusion criteria were audited before (n = 150) and after (n = 150) the two month EOV intervention period. The demographic characteristics of the audited patients are summarised in Table 4. There were no statistical differences between the two groups in age, sex, admitting specialty, or risk profile. The mean age of the groups was 70.8 (SD 14.4) and 72.4 (SD 13.9) years respectively. The majority of patients in both groups were admitted by a cardiologist (n = 91, 60% and n = 90, 60%). There was no significant difference in risk profile or contraindications to prophylaxis between the two patient groups (Table 2). The majority of patients were identified as being at a high risk of VTE (84% in the pre intervention group and 77% in the post intervention group).

**Table 4:** Characteristics of the audited patients

**Demographic**		**Pre intervention (n = 150)**	**Post intervention (n = 150)**	**P Value**
**Age**	Mean (SD)	70.8 (14.4)	72.4 (13.9)	0.33*
**Sex**	Number (%)			0.9^
Male		84 (56)	83 (55.3)	
Female		66 (44)	67 (44.7)	
**Admitting specialty**				0.98^
Cardiology		91 (61)	90 (60)	
Oncology		3 (2)	3 (2)	
Thoracic medicine		6 (4)	5 (3.3)	
Gastroenterology		11 (7.3)	8 (5.3)	
Nephrology		9 (6)	9 (6)	
Neurology		13 (8.7)	12 (8)	
Rheumatology		1 (0.3)	1 (0.3)	
Cardiac investigations		12 (8)	18 (12)	
Immunology		4 (2.7)	4 (2.7)	

Percentages may not add up to 100 due to missing data. *SD* Standard Deviation; *T test; ^Chi-square.

### Acceptability

Table 5 depicts the results of the participant and facilitator post intervention survey. Sixteen (94%) of the 17 participants who returned the post intervention survey reported that the EOV was effective or extremely effective at increasing their knowledge and 15 (88%) felt that it was effective or extremely effective at addressing their concerns about VTE prophylaxis for medical patients. The participants also agreed that the EOV was effective at providing information on the four key messages outlined in the study protocol: 16 (94%) participants reported that the EOV was effective or extremely effective at communicating the significance of VTE and the importance of VTE risk assessment; 15 (88%) agreed that the EOV was effective or extremely effective at providing information on selecting appropriate VTE prophylaxis; and 10 (59%) felt that the EOV was effective or extremely effective at providing information about ongoing monitoring. When asked how likely it was that they would participate in another EOV, 12 (71%) participants reported that it would be likely, or extremely likely. The same number (n = 12, 71%) felt that the EOV was likely, or extremely likely to influence their clinical practice. When the EOV facilitator was asked to rate the participants’ (n = 19) perceived interest, participation and comprehension in the EOV he reported that 11 (58%) participants had a high or very high level of interest; 13 (68%) had a high or very high level of participation; and 17 (89%) had a high or very high level of comprehension.

**Table 5:** Acceptability of the Educational Outreach Visit

**How effective was the Educational Outreach Visit in…**	**Extremely ineffective**	**Ineffective**	**Unsure**	**Effective**	**Extremely effective**
	**n (%)**	**n (%)**	**n (%)**	**n (%)**	**n (%)**
Increasing or refreshing your knowledge about VTE prophylaxis for medical patients?	0 (0)	1 (5.3)	0 (0)	11 (58)	5 (26)
Addressing concerns you have had about providing VTE prophylaxis to medical patients?	0 (0)	1 (5.3)	0 (0)	13 (68)	2 (11)
Providing information about the significance of VTE as a healthcare issue?	0 (0)	1 (5.3)	0 (0)	11 (58)	5 (26)
Providing information about VTE risk assessment for medical patients?	0 (0)	1 (5.3)	0 (0)	11 (58)	5 (26)
Providing information about selecting appropriate VTE prophylaxis for medical patients?	0 (0)	1 (5.3)	1 (5.3)	11 (58)	4 (21)
Providing information about the ongoing monitoring of patients risk and response to prophylaxis?	0 (0)	3 (16)	4 (21)	7 (37)	3 (16)
**How likely is it that…**	**Extremely unlikely**	**Unlikely**	**Unsure**	**Likely**	**Extremely likely**
You will participate in another educational program such as this one in the future?	1 (5.3)	0 (0)	3 (16)	11 (58)	2 (11)
This educational visit will influence your clinical practice?	1 (5.3)	0 (0)	3 (16)	11 (58)	2 (11)
**What was the participants perceived level of…**	**Very low**	**Low**	**Average**	**High**	**Very high**
Interest in the topic presented?	2 (11)	3 (16)	3 (16)	6 (32)	5 (26)
Participation during the visit?	1 (5.3)	1 (5.3)	4 (21)	3 (16)	10 (53)
Comprehension of the information provided?	0 (0)	0 (0)	2 (11)	7 (37)	10 (53)

Percentages may not add up to 100 due to missing data.

### Utility

Table 6 shows the descriptive data on the practical application and utility of the intervention. The median number of times it was necessary to make contact with participants to arrange the EOV was 3 (IQ range 1 to 4). The median time spent on each EOV was 92 minutes (IQ range 78 to 129) which was made up of time spent arranging the EOV (median 10 minutes, IQ range 10 to 20); customising the material (median 45 minutes, IQ range 45 to 60); waiting for the participant (median 5 minutes, IQ range 0–20) and conducting the EOV (median 15 minutes, IQ range 15 to 20). The majority of visits were conducted in the doctor’s office (n = 10, 53%). The remainder were held in the clinical area (n = 6, 32%); other public area (n = 2, 10%); or other private area (n = 1, 5%). At the completion of the EOV 15 (78%) of the 19 participants gave a verbal commitment to provide evidence-based prophylaxis. The facilitator’s self-reported adherence to all of the elements of the EOV protocol was 80% (IQ range 70–85).

**Table 6:** Utility of the educational outreach visit

**Measures**	**Median (IQ range)**
**Number of contacts needed to arrange the EOV**	
Number of contacts needed to arrange the visit	3 (1–4)
Number of cancelled visits prior to the visit	0
**Time spent arranging and conducting the EOV** (min)	
Time spent arranging the visit	20 (10–20)
Time spent customising material	45 (45–60)
Time spent waiting for the participant	5 (0–20)
Time spent with the participant during the visit	15 (15–20)
Time spent on interruptions	0
Total time spent on the visit	92 (78–129)
**Protocol adherence**	
Percent of protocol elements delivered to participant	80 (70–85)
**Location of the EOV**	**Number (%)**
Clinical area	6 (32)
Office	10 (53)
Other public area	2 (10)
Other private area	1 (5)
**Outcome of the EOV**	
Participant agreed to provide evidence-based prophylaxis	15 (79)

*IQ* Interquartile.

### Clinical impact

There was a significant improvement in the proportion of medical patients who received appropriate pharmacological VTE prophylaxis following the intervention (54% to 70%, 16% improvement, 95% CI 5 to 26, p = 0.004). Removing patients who were at lower risk of VTE from the analysis made no difference to the significance of the result (47% to 63%, 16% improvement, 95% CI 3 to 27, p = 0.01).

## Discussion

VTE is a major health and financial burden on the community [3]. Unfortunately, despite the availability of evidence-based guidelines, VTE prophylaxis is still frequently underutilised. Our study found that at baseline only 54% of medical patients were receiving evidence-based VTE prophylaxis. This confirms the evidence-practice gap described in the international literature [10-13]. Numerous strategies to improve VTE prevention in hospitalised patients have been studied but none have been successful at addressing all the barriers to the provision of evidence-based care [15-19].

The barriers to the provision of appropriate medical patient prophylaxis have been documented in a number [27,28] of recent studies. Known barriers include a lack of awareness of the importance of VTE prophylaxis and of the presence of evidence-based guidelines; a lack of knowledge on the indications for VTE prophylaxis and on appropriate prophylaxis options; and a lack of agreement and acceptance of current evidence-based recommendations [27,28]. EOVs acknowledge and address each participant’s barriers to change with the aim of facilitating increased compliance with evidence-based practice [20]. Few studies have examined the clinical impact of this intervention on the provision of VTE prophylaxis to medical patients and no previous studies have reported on its acceptability or utility.

Our results strongly suggest that EOVs are an acceptable implementation strategy for doctors working in the acute care setting. Nineteen (73%) of the 26 doctors eligible to participate agreed to receive an EOV. This was a greater than expected uptake given the established difficulty in providing hospital delivered education to senior doctors who, in the Australian private system, are consultant practitioners and not employees of the hospital [29]. It was also encouraging to find that following the intervention 71% (n = 12) of participants who provided feedback reported that they would participate in another EOV in the future.

By reporting descriptive data on the practical application and utility of the intervention we hope to provide valuable information for anyone wishing to use this intervention in an acute care hospital setting. Of particular note was the considerable time (92 minutes) required to organise, prepare and deliver each EOV. This study is one of a very few published studies to report the total time required for each EOV and the only study set in an acute care hospital setting.

Of the 19 participants who received the intervention 79% (n = 15) gave a verbal agreement to provide evidence-based VTE prophylaxis to their medical patients. Importantly, this commitment translated into a 16% (95% CI 5 to 26, p = 0.004) improvement in prophylaxis rates above baseline. This clinical impact is much larger than that reported in a Cochrane systematic review on the effectiveness of EOVs [21]. The review found that the median adjusted risk difference in compliance with prescribing practices was only 4.8% (IQ range 3.0% to 6.5%). The findings are similar, however, to two previous studies which used EOVs to improve doctors’ compliance with evidence-based VTE prevention practices in the acute care hospital setting. Roberts and Adams [25] observed a 14.2% (52.8% to 67%, p = 0.004) improvement in prophylaxis rates in medical patients while Grupper *et al.*[26] reported a 21% (29% to 50%, p < 0.001) improvement in a surgical population.

A limitation of our study was the use of a before-and-after design which may be subject to methodological limitations. There is some evidence to suggest that uncontrolled before and after studies over-estimate the effect of interventions [30]. Having only one post-implementation data point collected three months after the intervention means that we cannot know if the observed improvement in practice will be sustained or improved upon over time. The hospital plans to monitor the sustainability of the improvement as part of the hospitals ongoing quality systems. Future research is recommended that examines the clinical impact of EOVs on VTE prophylaxis using a cluster randomised controlled trial which includes an evaluation of the ongoing sustainability of the intervention.

## Conclusion

This study confirms that EOVs are effective at improving doctors’ provision of pharmacological VTE prophylaxis to hospitalised medical patients. In addition, it provides evidence of the acceptability of the intervention as an implementation strategy in the acute care setting, as well as valuable data on the practical application and utility of EOVs for those wishing to use this intervention in the future.

## Abbreviations

VTE: Venous thromboembolism; PE: Pulmonary embolism; DVT: Deep vein thrombosis; EOV: Educational outreach visit.

## Competing interests

The authors declare that they have no competing interests.

## Authors’ contributions

All authors contributed to the study concept, design and preparation of the manuscript. JD, AO and KW developed the intervention protocol. JD collected and analysed the data. All authors read and approved the final manuscript.

## Pre-publication history

The pre-publication history for this paper can be accessed here:

http://www.biomedcentral.com/1472-6963/13/398/prepub
